# St. Thomas' Hospital Reports

**Published:** 1891-12

**Authors:** 


*St. Thomas' Hospital Reports.* Edited by Dr. HADDEN and Mr. ANDERSON. Vol. XIX. Pp. xx.—463. London: J. & A. CHURCHILL. 1891.

In this volume we have a record of patient clinical observation, upon which a number of valuable papers on practical subjects are based.

Among many contributions, we may specially mention two articles on Intubation of the Larynx by Dr. W. W. Ord and Dr. H. G. Turney. Dr. Ord reports fourteen cases. He makes a point of introducing the gag in the right side of the mouth instead of in the left, the usual side, as he finds that when the gag is in the left side it is liable to interfere with the operator's right hand during the operation. We have never experienced any such difficulty; while we have found that the gag in the right angle of the mouth interferes with, or may become displaced by, the operator's left hand as it is passed down to the patient's larynx. Dr. Turney writes on the "Dangers of Intubation in Diphtheria." In the series of cases he has so faithfully recorded, he seems to have met with an unusual proportion of misadventures; but he states that he has "written on the principle that failures are often more valuable than successes," and his paper is full of practical service.

Dr. Michell Clarke reports a case of total transverse lesion of the cord, of especial interest as proving that muscular rigidities and the accompanying increased tendon reflexes may not occur when the connection of the brain with the cord is entirely cut off, and when there is further complete sclerosis of the pyramidal tracts.

A well-executed coloured plate, illustrating Mr. Richard Lake's Report of the Aural Department, renders the descriptions in the text very clear.

Dr. W. M. Ord writes on recent cases of myxœdema.

Taken altogether, this volume fully sustains the reputation of its predecessors.

